# Supplementary material for: Argonaute 2 targets viral transcripts but not genomes of RNA viruses during antiviral RNA interference in Drosophila
Source: PLoS Pathog. 2025 Feb 3;21(2):e1012184. doi: 10.1371/journal.ppat.1012184 (PMC11809787; doi:10.1371/journal.ppat.1012184)
Supplement: S1 Fig — Experimental design of dsGFP treatment in Hml-delta-GAL4, UAS-GFP flies in the left panel. Right panel shows GFP RNA levels at day 3 post treatment with dsGFP. Significant p value determined by two-tailed Mann–Whitney U-test is shown. (PDF) [file ppat.1012184.s001.pdf]

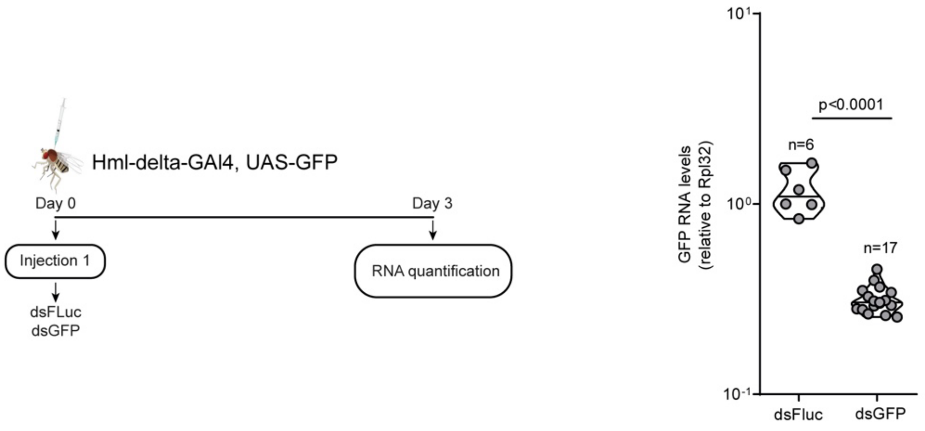

S1 Fig. Silencing of GFP expressed in transgenic flies. Experimental design of dsGFP treatment in *Hml-delta-GAL4, UAS-GFP* flies in the left panel. Right panel shows GFP RNA levels at day 3 post treatment with dsGFP. Significant *p* value determined by two-tailed Mann–Whitney U-test is shown.
