# Supplementary material for: Argonaute 2 targets viral transcripts but not genomes of RNA viruses during antiviral RNA interference in Drosophila
Source: PLoS Pathog. 2025 Feb 3;21(2):e1012184. doi: 10.1371/journal.ppat.1012184 (PMC11809787; doi:10.1371/journal.ppat.1012184)
Supplement: S2 Fig — (A) Immunofluorescence showing staining for AGO2, RpS15 and the VSV polymerase (RdRp) in infected and control S2 cells. Scale bar = 20 µm. (B) Structure of the Drosophila ribosome highlighting the subunits found to interact with AGO2 in control and VSV-infected cells (common interactants). Ribosomal subunits interacting with AGO2 in only one condition, mock or VSV-infected cells, are shown in different colors. The mRNA channel is indicated. (PDF) [file ppat.1012184.s002.pdf]

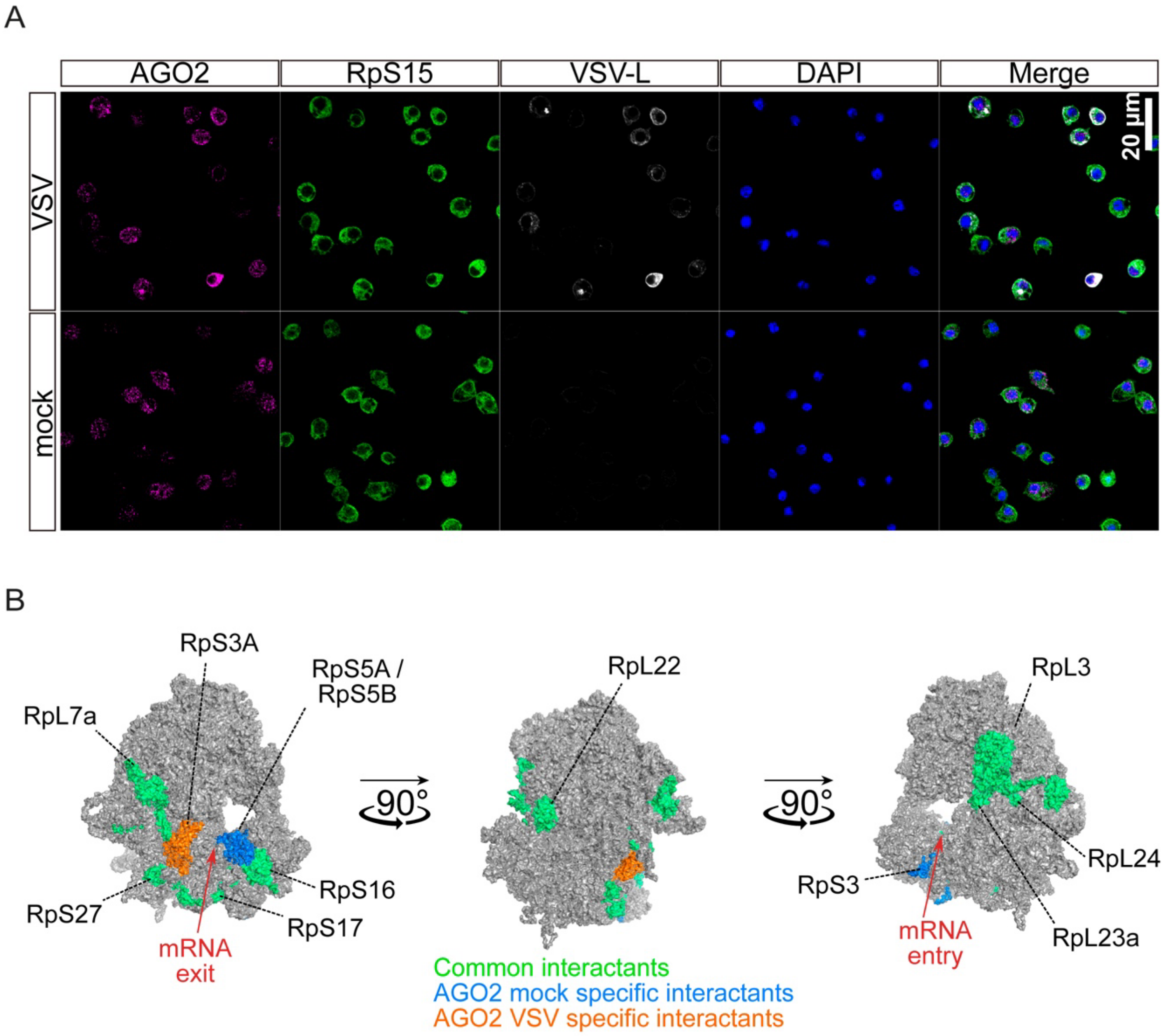

S2 Fig. AGO2 is mostly located in the cell cytoplasm in association with ribosomes. (A) Immunofluorescence showing staining for AGO2, RpS15 and the VSV polymerase (RdRp) in infected and control S2 cells. Scale bar = 20 µm. **(B)** Structure of the *Drosophila* ribosome highlighting the subunits found to interact with AGO2 in control and VSV-infected cells (common interactants). Ribosomal subunits interacting with AGO2 in only one condition, mock or VSV-infected cells, are shown in different colors. The mRNA channel is indicated.
