# Supplementary material for: Argonaute 2 targets viral transcripts but not genomes of RNA viruses during antiviral RNA interference in Drosophila
Source: PLoS Pathog. 2025 Feb 3;21(2):e1012184. doi: 10.1371/journal.ppat.1012184 (PMC11809787; doi:10.1371/journal.ppat.1012184)
Supplement: S1 Table — (PDF) [file ppat.1012184.s003.pdf]

S1 Table. Primers sequences utilized for RT-qPCR and dsRNAs constructions

Targets	Sequence
**Rpl32_F**	*GACGCTTCAAGGGACAGTATCTG*
**Rpl32_R**	*AAACGCGGTTCTGCATGAG*
**VSV-L_F**	*GGACATCCGGGGTAAGTTGG*
**VSV-L_R**	*CGTCCAGGGCTTTCAAGGAT*
**SINV-nsp2_F**	*CGTTCAGCTGCAAGACCA*
**SINV-nsp2_R**	*TACCGGCCGTGGCTAGTAT*
**SINV-sp_F**	*GTTCTAATGCGCTGCTGCTC*
**SINV-sp_R**	*CTGCCCTTTCAACAAGTGCC*
**GFP_F**	*CGACCACTACCAGCAGAACA*
**GFP_R**	*TCTCGTTGGGGTCTTTGC*
**dsVSV-N_F**	*TAATACGACTCACTATAGGGTCGGATGCTTCCAGAACCAG*
**dsVSV-N_R**	*TAATACGACTCACTATAGGGCAGAAGTGGAAGGCAGGGTT*
**dsSINV-nsp4_F**	*TAATACGACTCACTATAGGGGGTACCAGATGATGCCCACC*
**dsSINV-nsp4_R**	*TAATACGACTCACTATAGGGAGTCCAGTGTTGGCAGTTCA*
**dsGFP-F**	*TAATACGACTCACTATAGGGAGACCTGAAGTTCATCTGCACCA*
**dsGFP-F**	*ATTTAGGTGACACTATAGAAGTGGTTGTGGCGGATCTTGAAGT*
**dsFluc_F**	TAATACGACTCACTATAGGGAGAAACAATCCGGAAGCGACCAA
**dsFluc_R**	ATTTAGGTGACACTATAGAAGTGTGACTGGCGACGTAATCCAC
