# Supplementary material for: Argonaute 2 targets viral transcripts but not genomes of RNA viruses during antiviral RNA interference in Drosophila
Source: PLoS Pathog. 2025 Feb 3;21(2):e1012184. doi: 10.1371/journal.ppat.1012184 (PMC11809787; doi:10.1371/journal.ppat.1012184)
Supplement: Attachment — Submitted filename: Point_by_point_Response.pdf [file ppat.1012184.s007.pdf]

Point by point response to reviewers

Part I – Summary

Reviewer #1:

In this study, Silva et al. characterized the primary viral RNA targets of the Drosophila siRNA pathway during infections caused by VSV and SINV. Their data demonstrated that polyadenylated transcripts of VSV and SINV are the primary targets of silencing by the siRNA pathway during infection. They also stated that AGO2 copurifies with ribosomes, a process unaffected by virus infection. These findings enhance our understanding of the antiviral mechanism executed by RNAi silencing in Drosophila, particularly the specific viral RNA species targeted during RNA virus infection. Overall, the manuscript is well-written. However, the major findings are based on incomplete solid evidence. Additional experiments are needed to support the manuscript's major claims.

Reviewer #2:

This manuscript presents a series of experiments demonstrating that Drosophila Ago2-mediated antiviral RNAi (primarily) targets the polyA 'mRNA'-like transcripts, rather than genomes, of VSV (a -ssRNA virus) and SINV (a +ssRNA virus). The authors further argue that the strong association of Ago2 with a ribosomal fraction suggests a scanning mechanism whereby Ago2 interacts with transcripts as they are read by the ribosome.

The manuscript is well written, and I think the work is sound as far as it goes: the experiment is simple, but clever, and I could not immediately identify any faults in the approach or logic. My only concerns with scale and importance of the finding (is this big enough for PLoS Pathogens, even as a short report). Namely, the targeting of transcripts was already suspected, and previously shown in plants; a ribosomal association has been previously reported, but an artefactual association is not strongly ruled out here; the proposed scanning mechanism based on ribosomal-association is nice, but not really tested and remains hypothetical.

This is clearly an editorial decision, but on balance I think the paper is indeed suitable. Drosophila is a (the?) major model for antiviral RNAi in animals, the targeting of mRNAs is shown here quite

compellingly, and the mechanistic hypothesis is a good basis for further research. I have no doubt that this work will be read and cited by those working on antiviral RNAi and RNAi mechanisms more generally.

Response to Reviewers #1 and #2:

We thank reviewers for the positive comments and the concerns raised. We agree that our results showing the possible association of AGO2 with ribosomes were not particularly strong and did not go beyond what was already known, except for the analysis in virus infected cells. We have now included new experiments to address this issue, as summarized below:

1) We have performed immunofluorescence assays, which show that AGO2 is mostly in the cytoplasm of cells, overlapping mostly with the presence of ribosomes. Virus infection had some mild effects on AGO2 localization in this case.
2) We have performed mass spectrometry analysis of immunoprecipitated AGO2 from infected and non-infected cells compared to empty controls. This data shows clearly that AGO2 interacts with a subset of ribosomal proteins that are mostly located around the mRNA channel. Again, there were some mild changes in intercatants in virus infected cells, which may point to a change in function.

We think these new results show that AGO2 associates with ribosomes, with and without viral infection. Furthermore, our new data provides more mechanistic insights pointing to an interaction with ribosomal proteins located around the mRNA channel thus reinforcing the hypothesis of silencing associated with ribosome scanning.

Part II – Major Issues: Key Experiments Required for Acceptance

Reviewer #1:

1. Figure 1B: The authors injected VSV-WT at 5000 PFU/fly for the first experiment and VSV-GFP at 500 PFU/fly for the second. Intuitively, these viruses should replicate at higher levels given the 10X difference in viral load. However, the VSV-L RNA levels in these injected flies were similar to those in flies first injected with Mock, VSV-UV, and dsFluc. This result is difficult to understand.

Response to Reviewer:

This is an important point. Before doing the experiments, we performed dose responses and kinetics of replication for both VSV stocks and noticed that our VSV-WT grew at 10-fold lower efficiency compared to VSV-GFP. See figure below. Therefore, to provide equal amounts of replicating viruses, we used a 10-fold excess of VSV-WT.

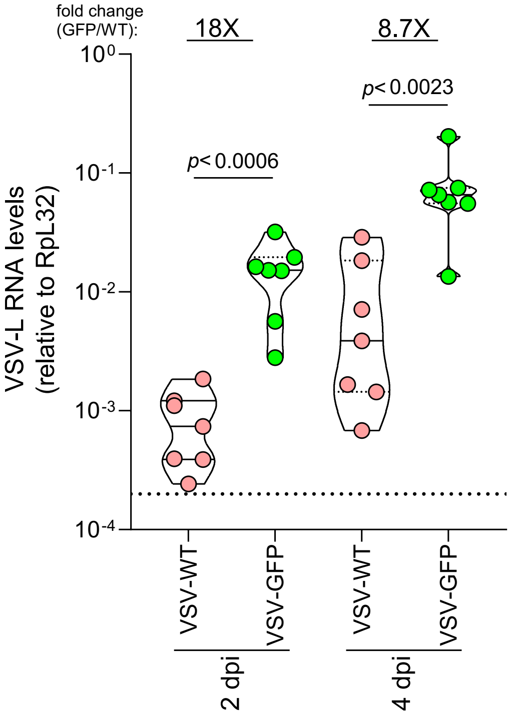

Figure Legend: Growth curves of VSV-WT and VSV-GFP in Canton S flies. Female adults were injected with 500 PFU of VSV-WT or VSV-GFP. 2- and 4-days post injection (dpi), individual flies were collected and total RNA extracted using TRIzol. VSV RNA genome was detected by RT-qPCR and viral loads normalized by RpL32. Significance was evaluated using Mann Whitney u test. Fold change was calculated by dividing viral load means of VSV-GFP/VSV-WT for each group as indicated at the top.

2. Figures 3A and 3B: The cell fractionation strategy described is too simplistic to provide detailed insights, even in the Methods section. According to the manuscript, the final P100 pellet contains

not only ribosomes but also other organelles and protein complexes. In Figure 3B, the authors used Rps15 as a ribosome marker. However, this result only proves that ribosomes are present in the P100 pellet; it does not confirm that AGO2 is associated with ribosomes. Additional experiments are needed to demonstrate AGO2's association with ribosomes.

Response to Reviewer:

We agree with the reviewer and have now include two independent sets of experiments to reinforce the association of AGO2 with ribosomes. These experiments have been described above in response to the comments in the summary section.

Part III – Minor Issues: Editorial and Data Presentation Modifications

Reviewer #1:

Figure 1C: Please verify the positions of the dsRNA and qPCR primers.

Response to Reviewer:

We thank the reviewer for these comments. The location and organization of the primers were indeed incorrect. We have edited the figure accordingly.

Reviewer #2:

My specific (minor) comments are listed below:

1. The title seems a little odd, as I would consider the dicing action of dicer2 to be a part of RNAi, and that does target the genome (during replication)

Response to Reviewer:

We agree with the reviewer and have adjusted the title to ‘Argonaute 2 targets viral transcripts but not genomes of RNA viruses during antiviral RNA interference in Drosophila’ thus better reflecting our observations.

2. Line 41: ‘a’ or ‘the’ major?

Response to Reviewer:

This has been corrected.

3. Line 48: this feels like an overclaim. Is there not some impact of Dicer2 activity on virus replication or copy number, regardless of the role of Ago2? I think I recall an Ago2 K/O paper that claimed Ago2 but not Dcr2 was dispensable for successful response to DCV (perhaps because of the VSRs encoded by DCV?). Although now I can’t find which paper …

Response to Reviewer:

I think the reviewer is referring to the manuscript by Eric Lai’s group (PMID: 19251644), where the authors show that dicing of Flock House virus replication intermediates was very efficient, but the siRNAs were not incorporated into AGO2. However, this was only observed in persistently infected S2 cells that harbor high levels of Flock House virus in culture. Even in this model, it is not clear whether Dicer-2 processing of the viral dsRNA is important for the control of the virus. In most other systems, Dicer-2 processing of genomes alone is not enough to contain RNA viruses as AGO2 deficient flies have basically the same phenotype as Dicer-2 deficiency. This makes sense since dsRNA processing requires access to virus-derived replication intermediates and seems very inefficient. Thus, considering our results, we feel that it is not an overstatement to say that silencing of transcripts accounts for the antiviral effect of the siRNA pathway.

4. L67. Do the authors think that their findings might bear on the patchy (along the genome) and biased (between the strands) distribution of viRNAs seen in many viruses? If so, it could be added to the discussion and siRNA properties introduced here.

Response to Reviewer:

Although the distribution of siRNAs along the viral genome is sometimes patchy, coding regions are always covered to some extent. It is possible that more efficient targeting siRNAs are selected, and this influences their distribution. Indeed, we observed before that, in the absence of AGO2 loading in R2D2 mutants, the distribution of siRNAs changes (PMID: 24009507). However, due to the space limitation of short reports, we do not have space to include this in the discussion.

5. L123-125. The small effect size suggests a very small proportion of the RNA is transcript rather than genome (am I correct in this interpretation?) – is that credible, from what is known of the replication of this virus?

Response to Reviewer:

Yes, the interpretation in correct. In our previous manuscript (PMID: 24009507), we showed that polyadenylated VSV RNAs represent a small fraction (~15-30% depending on the condition) of the total VSV RNA species in the cell.

6. L152-155 This observation, of the apparently highly localised nature of the scanning, is interesting and warrants more discussion and more emphasis. If I were to suggest expanding the paper with further experiments, this would be a good place to do it.

Response to Reviewer:

We agree that this is an important point, but we believe it is beyond the scope of this manuscript. This mechanism would require substantial further investigation that should be the subject of a separate manuscript.

7. L165-167 and elsewhere. The association with ribosomes is interesting, but for a high-copy entity like a ribosome, it is always a concern that this association may be spurious. It would be

very helpful to know what other (non-ribosomal) proteins are enriched int eh ribosomal fraction, and by how much. Ago1? Any other RNAi components?

Response to Reviewer:

We agree with the reviewer that ribosomes can easily be detected in many assays solely due to their immense abundance in the cell. However, as mentioned before, we have now included new experiments that reinforce the hypothesis of direct association between AGO2 and the ribosomes. In the mass spectrometry analysis of immunoprecipitated AGO2 that we performed, the enrichment results were obtained compared empty vector controls that should give us background contaminations. In this case, we have clearly observed enrichment of ribosomes in immunoprecipitated AGO2. Of note, we have used Dcr-2 for immunoprecipitations and this has not yielded enrichment for ribosomes, which reinforces the specificity of the association. This has not been included in this manuscript due to space, but we can do it at the editor's discretion.

8. L182-184 – I found something about the wording of this sentence to be opaque. I had to read it several times.

Response to Reviewer:

We have modified the sentence to make it more clear in the context of the discussion. It now reads 'Our new observations are also consistent with results suggesting that polyadenylated viral RNAs from VSV and SINV are preferentially silenced compared to viral genomic RNAs'.

9. Could the authors confirm that neither virus tested encodes a VASR that is functional in Drosophila, and could affect the experiment or its interpretation?

Response to Reviewer:

No VSRs encoded by VSV or SINV have been described and our previous results indicate that these viruses are unlikely to express active VSRs (PMID: 24009507). Both viruses are extremely sensitive to RNA interference and have a small RNA profile that is very similar to the obtained

from direct processing of dsRNA. Since they do not significantly affect Dicer-2 processing nor AGO2 silencing, it is safe to that they encode a VSR.

10. Line 234 – What is a canonical tube?

Response to Reviewer:

This was a typo. It has been corrected to conical tubes.

11. L271 ‘douncer’ is rather informal, if cute. The ‘Dounce homogeniser’ is named for Alexander Dounce

Response to Reviewer:

This has been corrected to tissue homogenizer.
